# Present and future of whole-body MRI in metastatic disease and myeloma: how and why you will do it

**DOI:** 10.1007/s00256-024-04723-2

**Published:** 2024-07-15

**Authors:** Frederic E. Lecouvet, Caroline Chabot, Lokmane Taihi, Thomas Kirchgesner, Perrine Triqueneaux, Jacques Malghem

**Affiliations:** Department of Medical Imaging, Institut de Recherche Expérimentale et Clinique (IREC), Institut du Cancer Roi Albert II, Cliniques Universitaires Saint Luc, Université Catholique de Louvain (UCL), Avenue Hippocrate, 10, B-1200 Brussels, Belgium

**Keywords:** Cancer, Imaging, MRI, Whole-body imaging, Bone, Metastasis, Multiple myeloma

## Abstract

Metastatic disease and myeloma present unique diagnostic challenges due to their multifocal nature. Accurate detection and staging are critical for determining appropriate treatment. Bone scintigraphy, skeletal radiographs and CT have long been the mainstay for the assessment of these diseases, but have limitations, including reduced sensitivity and radiation exposure. Whole-body MRI has emerged as a highly sensitive and radiation-free alternative imaging modality. Initially developed for skeletal screening, it has extended tumor screening to all organs, providing morphological and physiological information on tumor tissue. Along with PET/CT, whole-body MRI is now accepted for staging and response assessment in many malignancies. It is the first choice in an ever increasing number of cancers (such as myeloma, lobular breast cancer, advanced prostate cancer, myxoid liposarcoma, bone sarcoma, …). It has also been validated as the method of choice for cancer screening in patients with a predisposition to cancer and for staging cancers observed during pregnancy. The current and future challenges for WB-MRI are its availability facing this number of indications, and its acceptance by patients, radiologists and health authorities. Guidelines have been developed to optimize image acquisition and reading, assessment of lesion response to treatment, and to adapt examination designs to specific cancers. The implementation of 3D acquisition, Dixon method, and deep learning-based image optimization further improve the diagnostic performance of the technique and reduce examination durations. Whole-body MRI screening is feasible in less than 30 min. This article reviews validated indications, recent developments, growing acceptance, and future perspectives of whole-body MRI.

## Introduction

Since its introduction almost 20 years ago, whole-body MRI (WB-MRI) has become the imaging modality of choice in a growing number of indications [1, 2]. This article focuses on the present and future of oncologic applications of WB-MRI in bone metastases and multiple myeloma (MM), which represent the most common secondary and primary bone tumors in adults. It does not cover the role of WB-MRI in lymphoma and pediatric cancers and the numerous non-oncologic indications such as inflammatory involvement of bones, joints, or muscles [3].

WB-MRI has established itself as a credible alternative to techniques previously used in these disorders, such as radiographs, bone scintigraphy (BS), and computed tomography (CT). It rivals or outperforms positron emission tomography with computed tomography (PET/CT) in many indications and is the first-line imaging modality in several malignancies [4, 5].

From a technical standpoint, WB-MRI combines anatomical sequences, functional diffusion-weighted imaging (DWI) sequences, and quantitative measurements [6]. Specific guidelines provide cancer specific examination designs and recommendations for reading, reporting, and response assessment [7, 8]. The implementation of 3D acquisition and of the Dixon method and the growing role of artificial intelligence and of deep learning (DL)–based image optimization largely contribute to improve the diagnostic performance of the technique and reduce acquisition times [9, 10].

The capacity of WB-MRI for tumor screening has been extended from the skeleton to all organs, leading to an ever-increasing number of indications [11]. WB-MRI can now be recommended in a wide range of cancers for one-step staging, quantification of tumor burden, assessment of lesion response to treatment, and detection of residual disease.

This article reviews the basic technical principles of WB-MRI and recent developments to optimize the technique. It provides a practical approach to reading WB-MRI. It illustrates its established and emerging oncologic indications and discusses comparisons with historical and other “modern” imaging modalities. Finally, it presents our views on the challenges and promising future developments of WB-MRI.

## Technique

### Basic principles

WB-MRI relies on the sequential acquisition of high-resolution images of limited segments of the body, which are then merged by reconstruction software [12]. To reduce examination time, body coverage is often limited to an “eyes-to-thighs” study, which covers the skeletal areas affected by neoplastic bone marrow infiltration and most of the organs involved in systemic cancer spread [13].

Both 1.5 T and 3 T magnets currently provide very satisfactory results [14]. The simultaneous use of receiver coils specific to different regions of the body provide optimal signal-to-noise ratio and spatial resolution. This includes the use of the head and neck coils, tabletop coils for spine coverage, and torso phased array coils.

Most sequences are acquired in free-breathing mode, which is even recommended for DWI sequences, as it reduces motion artifacts and increases the signal-to-noise ratio. Some fast sequences (gradient echo 3DT1) can be acquired under apnea or with respiratory gating over the chest and abdomen.

Contrast-enhanced sequences are not performed routinely. Post-contrast sequences may be obtained on the liver or brain in certain cancers with a particular tropism for these organs or on the spine in symptomatic patients with suspected meningeal or epidural carcinomatosis. The brain, breast, and upper abdomen may be covered by specific post-contrast sequences in comprehensive evaluations of patients with syndromes predisposing to multiple cancers (see below).

A WB-MRI scan typically combines anatomical and “functional” diffusion-weighted imaging (DWI) sequences (Table 1).

**Table 1:**
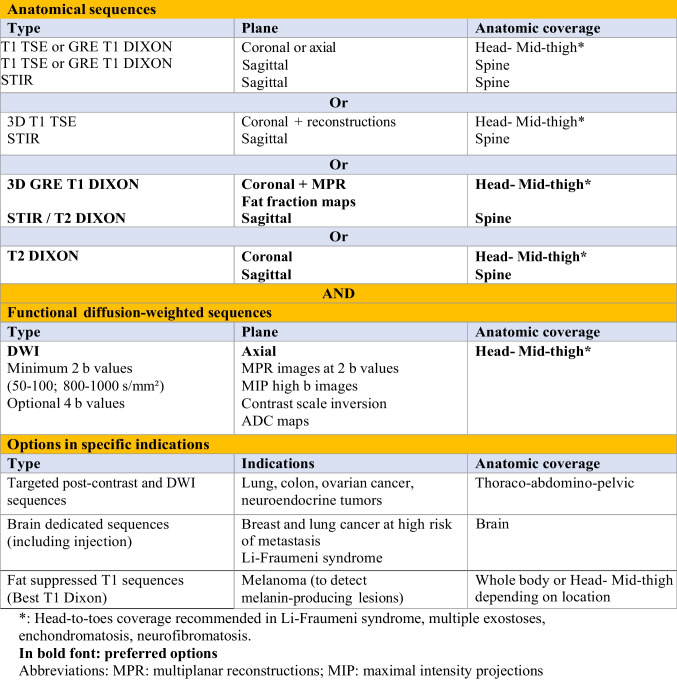
Whole-body MRI protocols in oncology

Recommendations have been published to harmonize and standardize content, quality, and reporting of WB-MRI performed in prostate cancer (MET-RADS-P) and multiple myeloma (My-RADS) [7, 8]. These recommendations also provide criteria for assessing the response of bone, lymph node, and visceral lesions based on qualitative and quantitative image analysis.

### Anatomical sequences

**Fig. 1:**
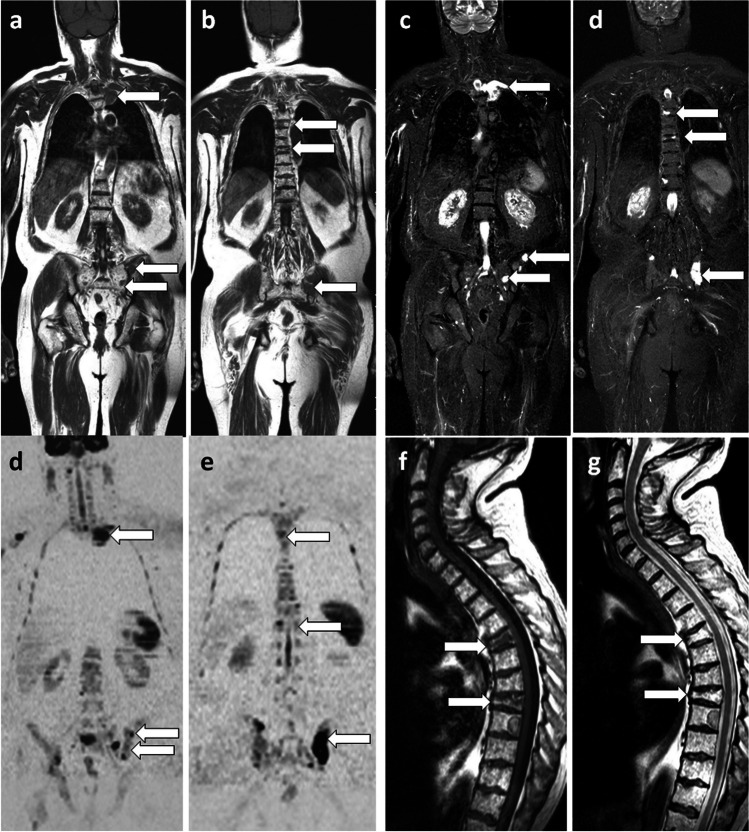
66-year-old man with newly diagnosed multiple myeloma: WB-MRI work-up. Coronal T1 (**a**,** b**), STIR (**c**, **d**) and DWI (*b* = 1000 s/mm^2^, inverted grayscale) DWI (**e**, **f**) images: multiple foci of bone marrow replacement are seen in the ribs, thoracolumbar spine and pelvis (arrows), indicating advanced disease requiring treatment. Sagittal T1 (**g**) and T2 (**h**) images of the spine: two pathologic vertebral compression fractures (arrows) are more clearly seen than on coronal sections

**Fig. 2:**
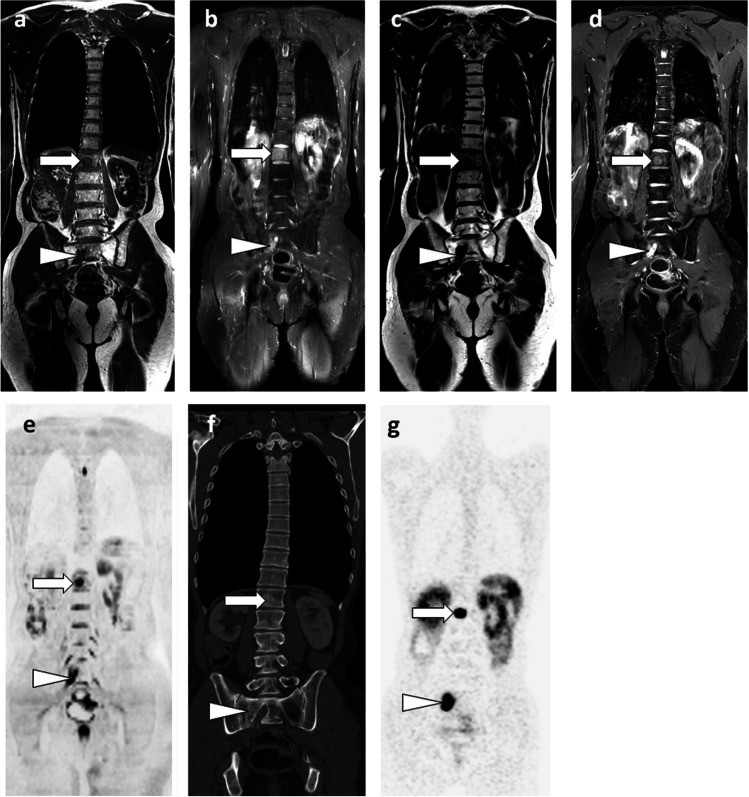
46-year-old man with newly diagnosed neuroendocrine cancer: comparison of T1 and STIR sequences with a single T2 Dixon sequence, DWI and PET/CT. **a**, **b** Coronal T1 and STIR WB-MRI sections show two metastases within the L1 vertebral body (arrow) and the right sacral wing (arrowhead). **c**, **d** Fat-only (FO) (**c**) and water-only (WO) (**d**) images of the corresponding FSE T2 Dixon sequence, show exactly the same lesions (in a single sequence). **e** Coronal high *b*-value (*b* = 1000 s/mm^2^, inverted grayscale) DWI sequence shows the same lesions. **f**, **g** Gallium-68 dotatate PET/CT (octreo-PET) show the same lesions, slightly sclerotic on CT (**f**) and showing intense tracer uptake (**g**)

Fat-sensitive T1-weighted sequences have been the historical reference for evaluating the bone marrow and its tumoral involvement. Optimization of bone lesion detection and extension of metastatic screening to extraskeletal organs have led to the addition of fluid-sensitive “T2-like” sequences, often acquired with fat signal suppression using various methods: chemical shift selective fat saturation (CHESS), inversion recovery (STIR), or the Dixon method (see below). The STIR sequence has been by far the most widely used in WB-MRI (Figs. 1 and 2). Finally, transverse T2-weighted fast spin echo (FSE) sequences are acquired to assess visceral and lymph node metastases [7].

These anatomical sequences are most often acquired as 2D images obtained in the coronal or axial planes. Sagittal sections of the spine can be acquired to optimize the delineation of vertebral lesions and their consequences on the spinal canal [2, 7] (Fig. 1).

### “Functional” sequences

DWI sequences provide information about cellularity and cell membrane integrity by assessing the diffusion properties of water molecules.

Technically, whole-body DWI consists of 2D MRI sequences acquired with high diffusion gradients and fat signal suppression, most commonly STIR. The *b*-factor, expressed in seconds per square millimeter, expresses the degree of diffusion weighting of the sequence and depends on the characteristics of the diffusion gradients. The use of two or more *b*-values allows a combination of anatomical information (on low *b*-value images), optimization of lesion detection (high *b*-value images), and calculation of the apparent diffusion coefficient (ADC). The lowest *b*-value (50 to 100 s/mm^2^) provides an image equivalent to a T2-weighted sequence. The high *b*-value (800 and 1000 s/mm^2^) images optimize contrast between lesions and their environment and are particularly useful for detecting bone lesions in areas that are difficult to study with anatomic sequences (ribs, shoulder girdle) and for detecting visceral lesions, especially liver or lung metastases, adenopathies, and peritoneal nodules. These high *b*-value images are often read in inverted grey scale windows, in a “BS or PET-like” fashion. DWI sequences are typically acquired in the axial plane, where they can be directly correlated with the usual acquisition plane of the more “conventional” cross-sectional imaging techniques, CT, and visceral MRI.

**Fig. 3:**
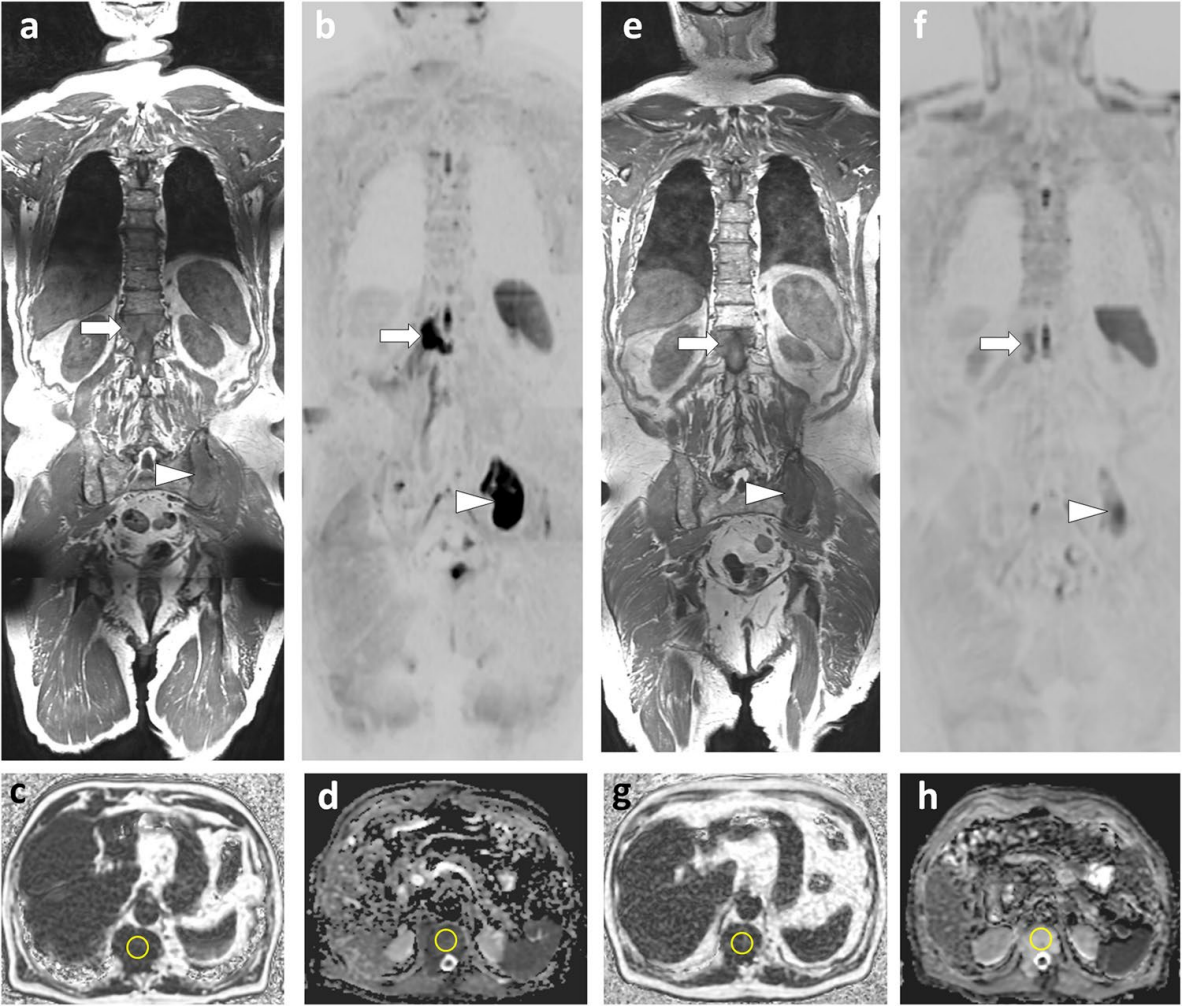
77-year-old man with multiple myeloma: WB-MRI studies at diagnosis and after treatment. **a**, **b** Coronal T1 (**a**) and high *b*-value (inverted grayscale window, *b* = 1000 s/mm^2^) DWI (**b**) sequences show a large lesion within the T12 vertebral body extending into the right paraspinal soft tissues (arrow). Another lesion is seen within the left posterior iliac crest (arrowhead). **c** Axial fat fraction (FF) map; measurement within the T12 body (circle) shows the absence of residual marrow fat (FF = 0%). **d** Axial ADC map; measurement within the T12 body (circle) shows increased ADC (750 mm^2^/s), indicating tumor infiltration. **e**–**h** Morphologic images and corresponding quantitative maps from the MRI follow-up examination performed two months later, after initiation of systemic treatment and irradiation of the lesions: regression in size and fading of the lesions on morphologic images (arrows and arrowheads in **e**, **f**). Measurements within the body of T12 show increased FF (16%) and ADC (1235 mm^2^/s) (circles), indicating lesion response to treatment

ADC maps are reconstructed for lesion characterization and evaluation of response, adding a “functional” dimension to the imaging study (Fig. 3).

In tumors involving solid organs such as the liver or prostate, this diffusion is restricted, “impeded,” due to the multiplication of membrane interfaces and the reduction of intracellular spaces, resulting in a decrease in ADC values (see below). In tumors involving the bone marrow, the water diffusivity is paradoxically increased, “enhanced,” at least in the early phase of tumor infiltration. This results from the limited diffusion capacity of water molecules within the normal bone marrow due to its composition of large cells, limited extracellular space, and predominant hydrophobic fatty content. This property, together with fat replacement, higher T1 and T2 relaxation times, and greater vascularity, explain the visibility of bone marrow lesions on high *b*-value DWI images.

### Multiparametric and quantitative imaging

Multiparametric MRI is defined as the combination of anatomical sequences and at least two “functional” quantitative sequences. In WB-MRI, the quantitative assessment of bone lesions relies on DWI sequences-based ADC maps and Dixon method–based fat fraction (FF) maps.

ADC maps are of great interest for lesion characterization and assessment of treatment response [6, 15, 16]. ADC is automatically calculated from signal intensities on diffusion-weighted images acquired at different *b*-values (Fig. 3).

WB-MRI sequences acquired in the Dixon mode are used to generate FF maps, which allow quantification of fat in the bone marrow and in bone lesions before and after treatment to assess response and appear to be a promising biomarker of that response [16] (Fig. 3).

### Optimization of WB-MRI examinations

Several approaches have been proposed to optimize the diagnostic effectiveness and minimize the duration of examinations.

#### Optimal sequence combination

Several studies have evaluated the diagnostic performance of individual anatomic and DWI sequences and their combinations in the detection of bone lesions [17]. These works suggest that (FSE or Dixon gradient-echo) T1 and DWI sequences are sufficient not only for the detection of bone metastases of prostate cancer, but also for the detection of lymph node and visceral involvement, with no additional benefit from a STIR sequence [18, 19].

#### Implementation of 3D acquisition

To avoid the repetition of anatomical sequences in 2D mode in multiple planes, 3D fast spin echo (FSE) sequences with very thin slices (1–1.5 mm isotropic voxels) have been developed to cover large fields of view [5, 9]. With their multiplanar capability, these sequences (cube, space) facilitate the detection of bone and lymph node metastases, avoid the need to acquire dedicated images of the spine, and minimize the risk of missing lesions due to partial volume effects associated with slice thickness in 2D acquisitions.

The acquisition of T1-weighted sequences using gradient echo (GE) and 3D modes further accelerates the acquisition of anatomical images and allows their acquisition under apnea. A study in patients with metastatic prostate cancer showed that a 3D T1 GE sequence acquired under apnea had similar performance to a 3D T1 FSE sequence in detecting bone and nodal metastases, while the acquisition time of this anatomical sequence was reduced from 14 to 1 min 20 s [20].

#### Use of the dixon method

The Dixon technique takes advantage of the different precession frequencies of water and fat protons to record the signal alternately over time when these protons are in phase (IP) and out of phase (OP) with each other. In addition to these IP and OP images, a mathematical calculation provides images that favor the fat only (FO) or water only (WO) signal, corresponding to fat-saturated images. Hence, the technique not only allows a reliable fat signal suppression (WO), but also provides anatomical sequences (IP), and sequences that maximize contrast between lesions and normal bone marrow (FO). It also allows the calculation of the FF maps.

The importance acquiring images with the Dixon mode in MM must be emphasized. Conventional FSE T1 sequences can miss myeloma lesions due to their spontaneous relatively high signal intensity and limited contrast with the surrounding high signal intensity bone marrow. FO images, which are available for T1 and T2 Dixon sequences, show the foci of bone marrow replacement much more clearly [10].

Paralleling preliminary observations in limited spine studies, a single T2 Dixon sequence covering the whole body plane can replace T1 and STIR sequences and may be sufficient as the anatomical component of WB-MRI for metastasis or myeloma screening (Fig. 2) [10, 21, 22]. A T2 Dixon sequence indeed combines T2-weighted images with fat signal suppression (WO, fluid-sensitive sequence, equivalent to STIR) and fat images (FO, fat-sensitive, providing information very similar to T1) [21]. The availability of 3D T2 Dixon sequences suggests that this approach will be sufficient as an anatomical sequence, offering not only multiplanar capability, fat- and fluid-sensitive sequences, but also morphological T2 images in all planes.

#### Acceleration techniques

Parallel imaging has allowed the development of the simultaneous multi-slice (SMS) acquisition technique for DWI sequences in WB-MRI. A study comparing SMS-DWI with usual DWI sequences showed that this approach allows metastatic detection and reliable ADC calculation [23].

These approaches to decrease acquisition times by means of conventional acceleration techniques (e.g., parallel imaging or compressed sensing) are now largely overshadowed by DL reconstruction algorithms [24]. These DL-based solutions (Air Recon DL, Deep Resolve) reduce image noise generated within the original raw data for all type of conventional and DWI sequences and revolutionize the image quality and acquisition times of WB-MRI [25–27]. The impact of the use of these algorithms on quantitative parameters such as ADC and FF maps deserves further evaluation [28].

## WB-MRI reading and lesion characterization

### Practical reading method

Morphological images (fat- and fluid-sensitive sequences, low and high *b*-value DWI images) and quantitative ADC and FF maps are read on PACS workstations by “linking” and browsing them in the different planes using multiplanar registration and reformatting tools. These images are complementary and allow detection, localization, and characterization of lesions by comparing their appearance on different sequences and measuring ADC and FF. Maximum intensity projection (MIP) views of high *b*-value DWI sequences, displayed as 3D MIP images, rotating around a central cranio-caudal axis using inverted grayscale window, provide global at-a-glance disease assessment [7].

### Lesion detection: morphological and quantitative approaches

#### Bone lesions

On T1-weighted (fat-sensitive) images, the replacement of normal bone marrow by neoplastic cells causes a focal or diffuse decrease in the bone marrow signal intensity, which becomes lower than the signal of muscles and intervertebral discs [2, 29]. On T2 Dixon WO, fat-suppressed T2, or STIR (fluid-sensitive) images, the signal of bone lesions is variable, depending on the phenotype of the lesion, which may be more or less hydrated (higher signal) or sclerotic (lower signal). On DWI sequences, metastatic bone disease is characterized by focal or diffuse high signal abnormalities on high *b*-value images, with ADC values higher than those of normal bone marrow. ADC values in tumor tissue are typically greater than 1.2–1.4 × 10^−3^ mm^2^/s. A threshold ADC value of 0.655 × 10^−3^ mm^2^/s has been suggested to differentiate normal bone marrow from bone marrow infiltration by cancer cells [30].

Some pitfalls of DWI images need to be emphasized.

Although high *b*-value DWI images have a high sensitivity for lesion detection, their specificity is limited and correlation with ADC maps and anatomical sequences is essential to avoid false-positive observations. Certain benign lesions (hemangiomas, benign fractures, periarticular degenerative edema-like lesions, juxtadiscal fibrovascular endplate changes or Modic type 1 changes, focal or drug-induced diffuse hyperplasia of hematopoietic bone marrow) may cause abnormal signal on these images [31, 32]. Demonstration of the presence of fat within these lesions, facilitated by the Dixon sequence, is key to identifying these benign conditions.

ADC measurements also allow differentiation of viable tumors (which have lower ADC values) from hemangiomas (which have higher ADC values) and from necrotic metastases or treated myeloma foci (which may have high signal on high *b*-value images due to the “T2 shine through” phenomenon, but have much higher ADC values than viable tumors) [33].

False-negative DWI results can be observed in case of hypercellular bone marrow, which reduces the contrast between metastases and the surrounding marrow, and in cases of densely sclerotic metastases [2].

#### Lymph node and visceral lesions

Detection of malignant lymph nodes on WB-MRI is challenging, even on DWI sequences, because both normal lymph nodes and tumoral adenopathies have high signal on high *b*-value images due to their high cellularity. As with conventional CT, size remains the main criterion for the diagnosis of abnormal lymph nodes, which has known limitations: many tumoral lymph nodes are infracentimetric, and supracentimetric lymph nodes may not be metastatic [34].

Hepatic and pulmonary metastases present as lesions with high signal on high *b*-value DWI images. Their diagnosis is complemented by T2-weighted anatomical sequences. Studies have shown the high sensitivity of WB-MRI for detecting these visceral lesions compared with CT and FDG PET/CT [35].

### Assessment of tumor response

Imaging assessment of treatment response is a major expectation in oncology. Bone metastases, long considered “non measurable” due to the limitations of BS and CT, can now be assessed with WB-MRI and PET/CT [36]. On anatomic sequences and high *b*-value DWI, response assessment is based on the observation of changes in number, size, and signal of lesions, extending to the bone marrow the approach used for visceral lesions according to the RECIST (Response Evaluation Criteria in Solid tumors) recommendations [37].

Measurements of FF and ADC allow refinement and quantification of treatment response. Responding lesions show a decrease in signal on high *b*-value DWI images and an increase in ADC and FF. Progressive lesions show high signal on high *b*-value DWI sequences and decrease in ADC and FF (Fig. 3). These response criteria have been validated in both metastatic disease and MM, with correlations to histologic findings and patient survival [15, 38]. After intensive treatment, WB-MRI is used to detect “imaging minimal residual disease,” which also correlates with shorter survival [39].

Thresholding techniques applied to DWI sequences allow quantification of the total tumor burden and its monitoring over time to assess response to treatment [40].

## Indications, diagnostic performance, and comparisons with other techniques

WB-MRI was first used to detect bone involvement in cancers with a particular skeletal tropism in their metastatic spread, such as breast and prostate cancer, and in hematologic cancers that exclusively or frequently affect bones, such as MM [41].

Its diagnostic performance is superior to that of BS and CT for detecting bone metastases and to that of skeletal radiographs for myeloma staging. With the introduction of DWI, it has demonstrated its ability to detect extra-skeletal metastases and to provide “all-organ” staging, extending its indications to many other cancers (Table 2).

**Table 2:**
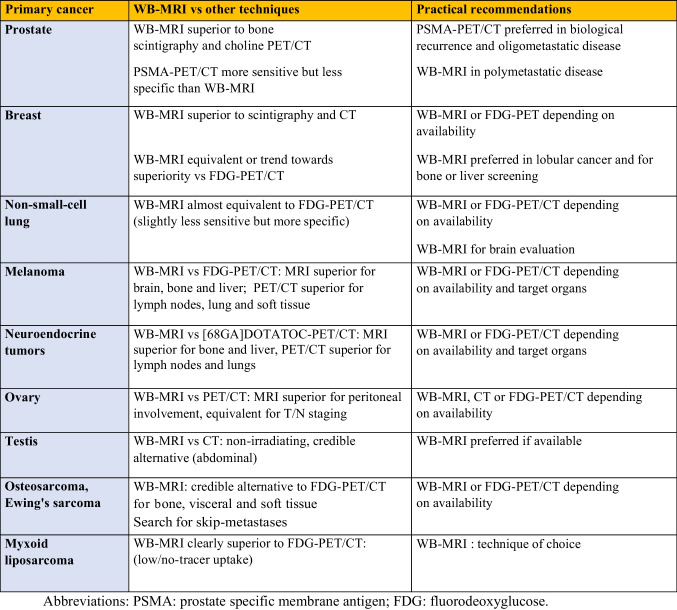
Indications for WB-MRI in metastatic screening and comparison to other techniques

### Metastatic cancers

**Fig. 4:**
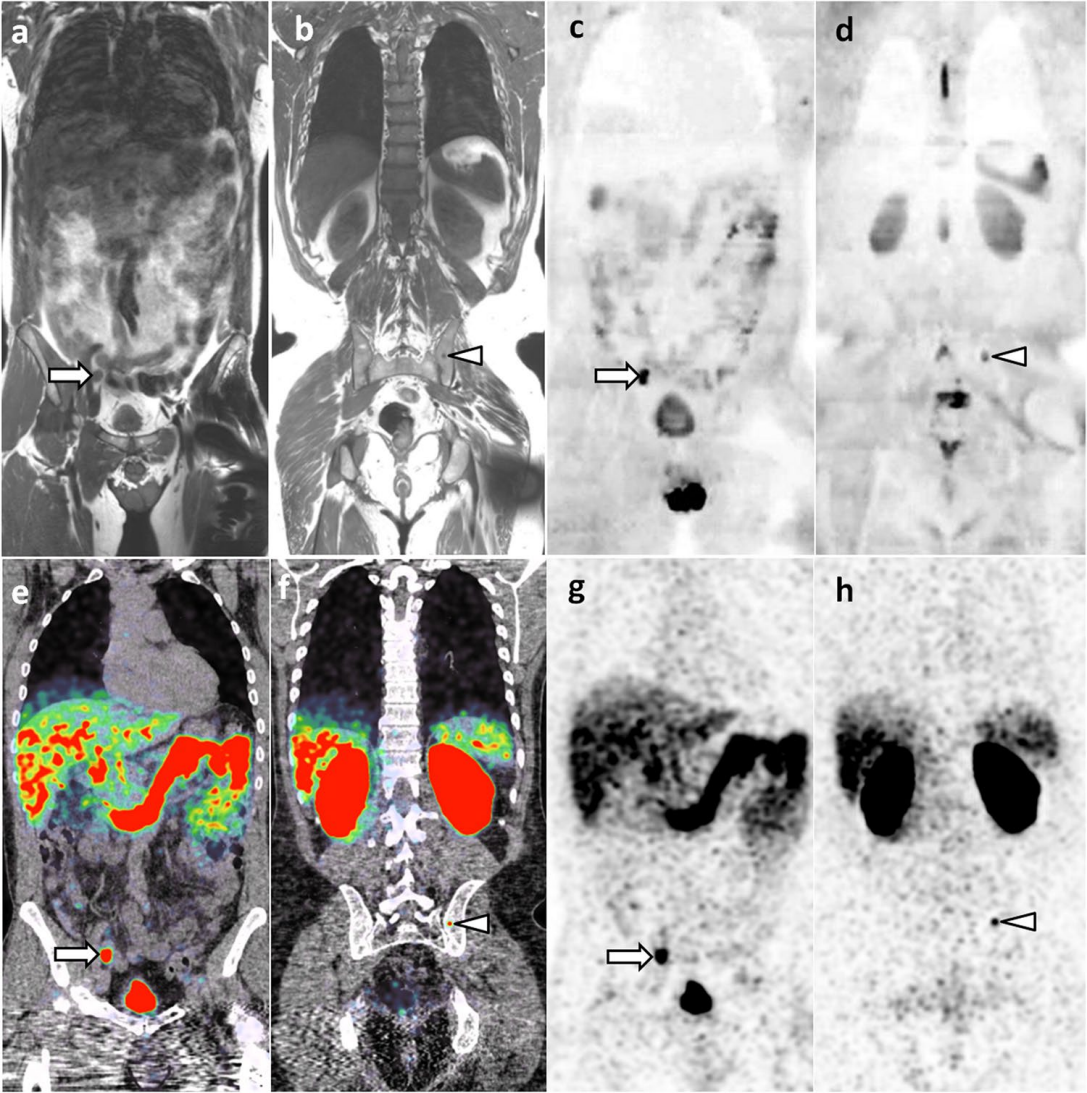
69-year-old man with newly diagnosed prostate cancer: comparison of WB-MRI and PSMA-PET/CT findings. **a**–**d** Coronal WB-MRI T1- (**a**, **b**) and high *b*-value (inverted grayscale window, *b* = 1000 s/mm^2^) DWI-weighted (**c**, **d**) images show right iliac lymph node metastasis (arrow) and left iliac bone metastasis (arrowhead). **e**–**h** Corresponding coronal images of PSMA-PET/CT (PET/CT fusion (**e**, **f**) and PET (**g**, **h**)) show the same lymph node (arrow) and bone (arrowhead) metastases

In prostate cancer, WB-MRI is more effective than the combination of BS and thoraco-abdomino-pelvic CT for lymph node (N), bone, and visceral (M) staging, allowing this staging to be made in a single step [11]. The performance of MRI seems to be superior to that of 11C- or 18F-labeled choline PET/CT [42]. The best technique currently available in nuclear medicine is prostate-specific membrane antigen (PSMA) PET/CT. Few studies directly compared WB-MRI and PSMA PET/CT (Fig. 4). A recent study showed a higher sensitivity (90–96%), but a lower specificity (70–90%), of PSMA PET/CT compared to WB-MRI (sensitivity 43–80%; specificity 96%) [43]. The choice between these techniques may depend on the stage of the disease. PSMA PET/CT is preferred at the time of biological recurrence (rising PSA) and for the diagnosis of oligometastases (see below). WB-MRI may be suggested as an alternative for the expected diagnosis of polymetastatic disease based on clinical and biological data, in the case of equivocal results not only from BS or CT but also from PSMA-PET/CT (numerous false positives and false negatives due to lack of PSMA expression by cancer cells), in advanced disease (castration-resistant cancer) prior to new therapy to assess disease response (including Lutetium 177-PSMA treatment), and in centers lacking PSMA-PET/CT [44].

**Fig. 5:**
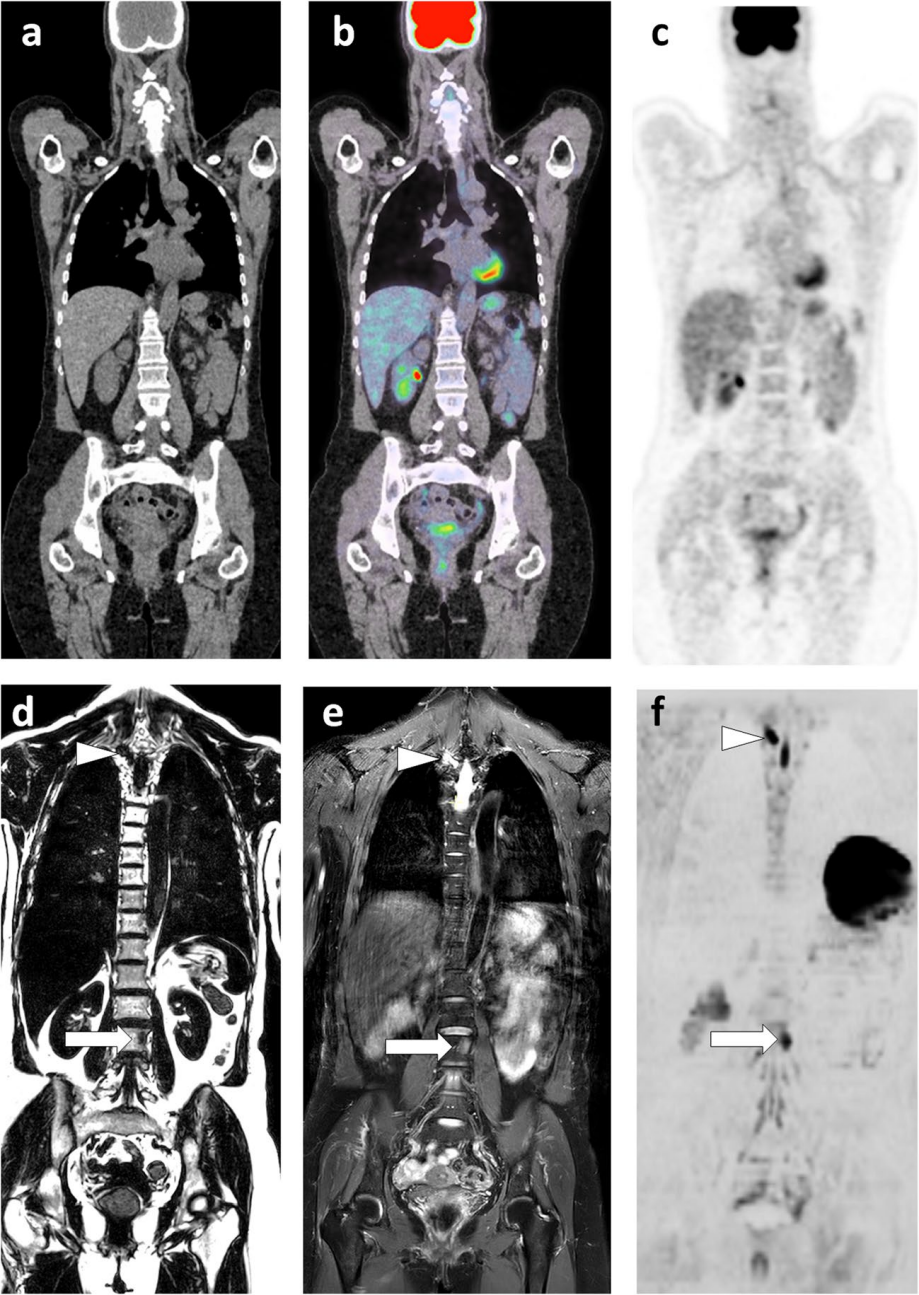
63-year-old woman with lobular carcinoma of the breast and increased tumor marker: comparison of WB-MRI and FDG-PET/CT**.** a–c Coronal PET/CT images (CT, fusion, PET): absence of evident lesion. **d**–**f** Corresponding coronal WB-MRI sections: fat-only (FO) (**d**) and water-only (WO) (**f**) images of a T2-weighted Dixon sequence, and coronal high *b*-value (inverted grayscale window, *b* = 1000 s/mm^2^) DWI image (**f**): bone marrow foci (metastases) at the left corporeo-pedicular junction of L3 (arrow) and the right transverse process of T3 (arrowhead). In total, three metastases were identified by this examination

In breast cancer patients at risk of metastasis spread, WB-MRI and fluorodeoxyglucose PET/CT (FDG PET/CT) are currently the most effective techniques for detecting metastases [45]. The choice between these techniques must be guided by the histologic and molecular characteristics of the primary tumor and the expected tropism of the metastases. PET/CT, which performs poorly in lobular carcinoma due to lack of glucose avidity of the cancer cells, should be replaced by WB-MRI in this indication [35, 46] (Fig. 5). WB-MRI with DWI sequences is the preferred technique in patients with predominantly or exclusively bone and liver metastases, including in late disease stages, when tissue changes associated with multiple lines of therapy make it difficult to detect viable tumor tissue. WB-MRI is much earlier and more discriminating than BS and CT in detecting metastatic response or progression on treatment [47].

Concerning other cancers, WB-MRI can replace conventional multimodality staging based on CT and PET in lung and colorectal cancers, reducing the number of examinations, time, and cost of staging [48–50].

In malignant melanoma, both PET/CT and WB-MRI have high diagnostic accuracy for detecting metastases, although their performance varies in different organs. WB-MRI performs better for liver, skeletal, and brain metastases, whereas PET/CT performs better for lymph node, lung, and soft tissue metastases [51, 52]. The value of T1-weighted sequences acquired in Dixon mode (in addition to diffusion sequences) should be emphasized, as WO (fat suppressed) images are highly sensitive to the presence of melanin [4].

For neuroendocrine tumors, WB-MRI with DWI is a reliable alternative to [68GA]DOTATOC-PET/CT. These techniques have similar performance in detecting metastases, but their performance differs by organ, with WB-MRI being superior for liver and bone metastases and PET/CT being superior for detecting lymph node and lung lesions [53] (Fig. 2).

**Fig. 6:**
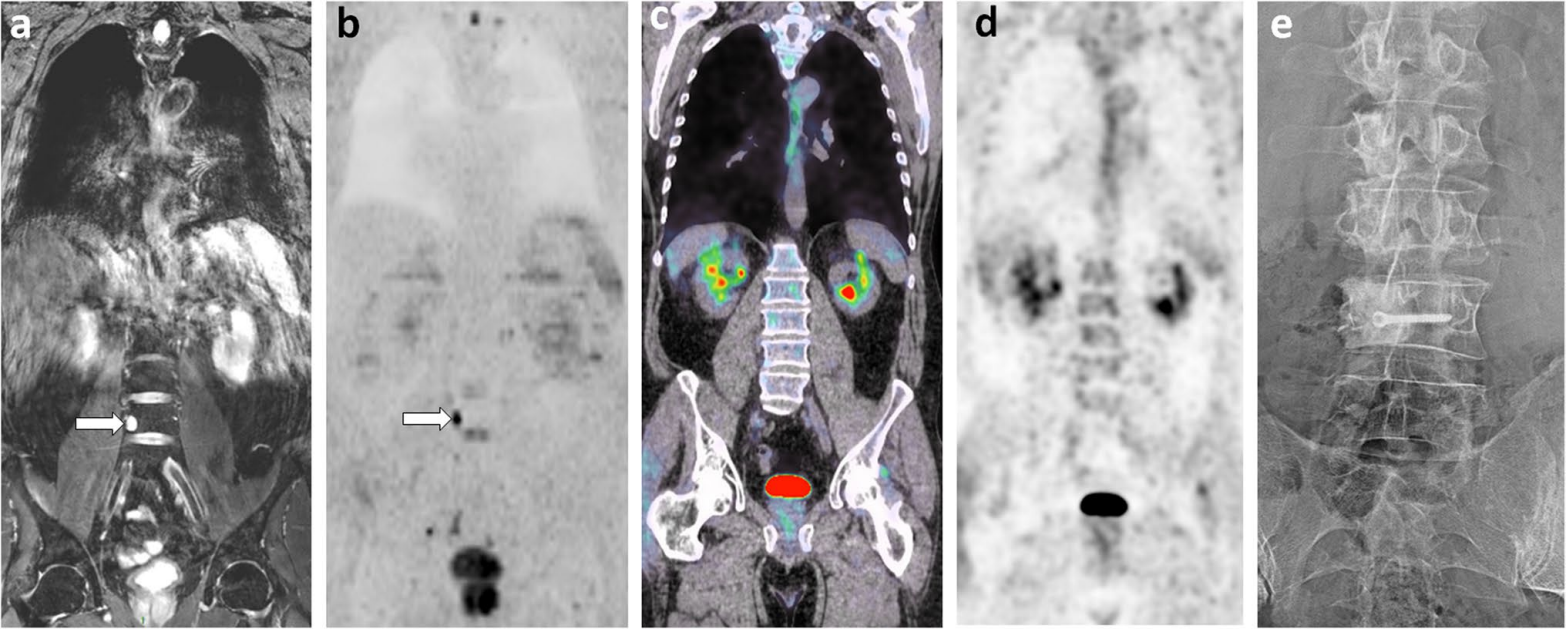
63-year-old man, 2-year imaging follow-up after resection of a myxoid liposarcoma of the thigh, non-metastatic at diagnosis: comparison of WB-MRI and PET/CT. **a**, **b** Coronal water-only (WO) T2-weighted (from a Dixon T2 sequence (**a**)) and high *b*-value DWI (**b** = 1000 s/mm^2^; inverted grayscale window) (**b**) images: Unique focus of bone marrow replacement is seen on the right aspect of the L4 body, with high signal intensity on T2 (myxoid content) and DWI (arrows). **c**, **d** Concurrent coronal PET/CT images (PET/CT fusion (**c**); PET (**d**)): no lesion is seen. **e** Radiograph performed after resection of the right portion of the L4 vertebral body: bone allograft and screw are seen within the site of partial vertebrectomy in the context of surgical resection of an “oligometastasis”. Pathology of the resected specimen confirmed complete resection of a metastasis from the primary myxoid liposarcoma

Myxoid liposarcoma is a soft tissue cancer that tends to metastasize to unusual sites, especially skeletal, but also visceral. FDG-PET/CT is not reliable for staging this disease and detecting metastases. Conversely, several studies have shown that WB-MRI is highly effective in this indication, making it the imaging modality of choice [54, 55] (Fig. 6).

In osteosarcoma and Ewing’s sarcoma, WB-MRI provides a comprehensive assessment of the entire skeleton and soft tissues, facilitating early detection of distant metastases as well as skip metastases within the involved bone.

WB-MRI is also gaining indications in cancers that do not often metastasize to bone. In ovarian cancer, it can replace CT and PET/CT [56]. In testicular cancer, WB-MRI with DWI is a non-irradiating, highly effective alternative to CT for detecting metastases — which mainly affect the abdominal lymph nodes — and for monitoring these young patients [57, 58].

### Multiple myeloma (MM)

In MM, the demonstration of lytic bone lesions, along with renal failure, anemia, and hypercalcemia, indicates advanced disease requiring aggressive treatment. For decades, extensive radiographic skeletal surveys were used to detect lytic lesions [59]. Low-dose whole-body CT has improved lesion detection and has been proposed as a first-line imaging modality [60]. However, its inability to assess lesion response to treatment limits its value. FDG-PET/CT is highly effective, based on the detection of osteolysis by CT and metabolic activity of MM foci by PET [61]. The technique is also effective in assessing response and detecting residual disease after treatment. WB-MRI allows detection of focal or diffuse bone marrow infiltration in MM, evaluation of response to treatment, and detection of macroscopic residual disease after treatment [39].

CT, WB-MRI, and PET/CT are now included in guidelines for the diagnosis and staging management of MM. WB-MRI has been introduced in the recommendations of the International Myeloma Working Group (IMWG), which define advanced MM by the observation of at least one evident bone marrow lesion greater than 5 mm on MRI [62]. The UK National Institute for Health and Care Excellence recommendations (NICE) guidelines position WB-MRI as the first-line imaging modality to be performed in suspected MM, preferable to radiographs, CT, and FDG-PET/CT for cost-effectiveness reasons [63]. MRI is also recommended for the management of patients with monoclonal gammopathy of undetermined significance (MGUS) at risk of progression, solitary plasmacytoma, and indolent MM [64].

WB-MRI, which is sensitive to bone marrow infiltration, has shown better performance in identifying bone involvement than multidetector CT, which requires the presence of osteolysis [65].

Studies comparing the diagnostic performance of WB-MRI and FDG-PET/CT in the same population are limited [66]. MRI generally performs better in detecting small focal lesions and diffuse bone marrow infiltration, although it must be kept in mind that MRI may face limitations in diagnosing low-grade diffuse bone marrow infiltration [29, 39, 67]. On the other hand, FDG PET/CT fails in 10–20% of MM that do not metabolize FDG in the absence or underexpression of hexokinase 2 [68]. MRI is also the method of choice to detect and characterize vertebral compression fractures, which may be caused by macroscopic MM foci or by the disease-related osteoporosis, and to assess their consequences on the spinal canal [69].

Both WB-MRI and PET/CT allow the detection of extramedullary bone involvement, which has become increasingly common with therapeutic advances and the proliferation of treatment lines and has a negative prognostic value. WB-MRI and FDG-PET/CT are also used to detect “macroscopic” imaging residual disease after treatment, which has a cardinal prognostic value [39, 70, 71].

Interestingly, WB-MRI is now preferred over CT or PET/CT by many teams around the world. While only 9.8% of UK centers used WB-MRI as a first-line modality for MM in 2018, despite national guidelines, and while the IMWG advocates low-dose CT or PET/CT for first-line imaging, a recent non-exhaustive survey shows that a large number of centers worldwide are now performing WB-MRI as a first-line modality [62, 72] (Table 3).

**Table 3:**
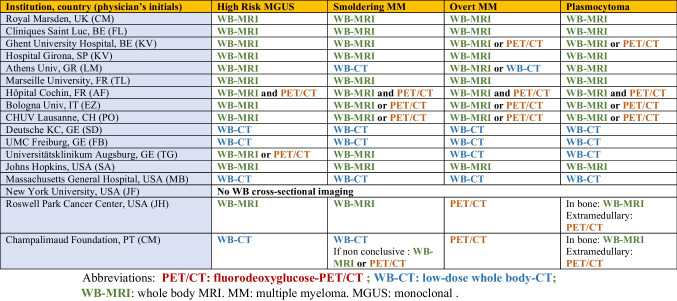
Current first-line imaging in myeloma and other plasma cell disorders at academic centers around the world. Results of a limited survey conducted in preparation for refresher courses on imaging in multiple myeloma to be given at the International Skeletal Society Annual Meetings in 2023 (London) and 2024 (Montreal)

## Particular conditions and emerging indications

### Oligometastatic cancer

With few exceptions, solid tumors that have reached a polymetastatic stage are considered incurable. The concept of “oligometastatic disease” postulates that there is an intermediate tumor state between localized cancer and polymetastatic disease [73]. This state could benefit from specific aggressive treatment (targeted radiotherapy or surgical resection) to achieve disease remission, delay complications and subsequent lines of therapy, and improve survival. Preliminary studies have shown improved survival when radical treatment of the primary tumor is combined with ablative therapy targeting metastases. This approach requires optimized detection and localization of all metastatic sites, for which the critical role of “modern” imaging such as WB-MRI with DWI sequences and PET/CT has been highlighted [45, 74, 75](Figs. 4 and 5).

###  Cancer and pregnancy

The occurrence of cancer during pregnancy is not uncommon (100–150 cases/100,000 pregnancies) [76]. The most common cancers occurring during pregnancy are breast cancer (41%), lymphoma (12%), cervical cancer (10%), leukemia (8%), ovarian cancer (7%), and other cancers (gastrointestinal, melanoma, thyroid, etc.) (22%).

Its non-irradiating nature and diagnostic performance make WB-MRI the imaging modality of choice for clinically detected malignancies during pregnancy [77]. It can be used to perform a one-step staging, and to evaluate the response to therapy, as many of these cancers can be treated during pregnancy [78].

WB-MRI is also used to detect, localize, and stage asymptomatic primary cancers in an increasing number of pregnant women whose cancer is detected by non-invasive prenatal testing (NIPT) [79]. This test is primarily used to detect abnormal circulating extracellular DNA in the mother’s blood, which indicates fetal malformations (trisomies). However, the technique can also detect tumoral DNA originating from an occult cancer in the mother.

### Predisposition to cancer

WB-MRI is recommended for the evaluation and follow-up of patients with musculoskeletal lesions at risk for malignant transformation and in patients with a genetic predisposition to cancer.

These include peripheral nerve cancers (neurofibromatosis type 1 (NF1), neurofibromatosis type 2 (NF2), schwannomatosis, etc.). WB-MRI is recommended in NF1 patients as a screening tool for the presence of plexiform neurofibromas, which may be a risk factor for the future development of malignant peripheral nerve sheath tumors [80, 81]. The subsequent role in detecting malignant transformation of lesions is discussed and compared with that of FDG PET/CT.

WB-MRI is recommended for early detection of malignant transformation in multiple exostoses and enchondromatosis. It is recommended in pediatric oncology for many childhood cancer predisposition syndromes [82].

Li-Fraumeni syndrome is an autosomal dominant cancer predisposition syndrome associated with abnormalities in the p53 tumor suppressor gene [83]. The most common cancers are of soft tissue (muscle, especially rhabdomyosarcoma), bone (osteosarcoma), breast, skin, colon, pancreas, adrenal, brain, and blood (leukemia). The risk of developing multiple cancers at the same time is also significant. Patients have a cumulative cancer risk of 90% by the age of 60. In addition, repeated exposure to irradiating diagnostic procedures further increases the risk of secondary cancers compared with the general population [6]. According to a landmark meta-analysis of 578 Li-Fraumeni patients, routine MRI detected 42 cancers in 39 individuals, a detection rate of 7% [84]. Current recommendations for patient surveillance include annual WB-MRI as part of a comprehensive evaluation that includes a physical examination, biological tests, other imaging studies, and colonoscopy. This surveillance improves survival in patients who undergo it compared to those who do not [85].

## Challenges and perspectives

WB-MRI has become the standard of care according to professional society guidelines and expert recommendations, e.g., in lobular breast cancer, myxoid liposarcoma, neurofibromatosis, and other cancer predisposition syndromes. It is on the same path in many other cancers. This will inevitably lead to its implementation in radiology departments with subsequent need for local competent radiologists. Acceptance by radiology departments and hospital authorities will largely depend on the availability of reimbursement codes, which exist in the UK and are currently being discussed with health authorities in several countries. An interim solution has been proposed using a combination of billing codes for chest, abdominal, and pelvic MRI [6].

WB-MRI reading requires a wide range of reader expertise, from skeletal radiology to visceral imaging, which can be addressed through collaborative subspeciality reading. These skills are close to those of PET/CT readers and suggest the emergence of “onco-imaging” as a true medical subspecialty at the interface of radiology and nuclear medicine.

The large number of images produced in WB-MRI requires considerable reading time and implies a risk of incidental findings and of missing some lesions. Software tools have been developed to aid detection, quantify “total tumor burden,” and extract quantitative information from FF and ADC maps to facilitate the management of these examinations [86].

Examination time, long considered prohibitive in radiology departments, has been significantly reduced. The combination of T2 Dixon and DWI sequences acquired with DL reconstructions allows WB-MRI examinations to be performed in less than 30 min. This facilitates patient and radiology department acceptance. The absence of radiation (particularly relevant for younger patients) and lower costs than PET/CT also increase the preference for WB-MRI [63].

Patient acceptance and even preference for WB-MRI over CT and multimodality staging have been repeatedly reported [87, 88].

Next developments will include assessment of the value of quantitative WB-MRI parameters as biomarkers, the reproducibility of WB-MRI response criteria, their ability to allow timely treatment switching, and their prognostic value in terms of patient outcomes.

WB-MRI will also benefit from the introduction of pseudo-CT MRI sequences capable of detecting osteolysis or sclerosis, important information that is missing from conventional MRI sequences and that is critical in the evaluation of multiple myeloma, and of the fracture risk and lesion response to treatment in metastatic disease.

## Conclusion

By combining anatomical and functional information, WB-MRI offers an inherently hybrid approach to optimize lesion detection.

Together with PET/CT, WB-MRI has become the technique of choice for early diagnosis, single-step skeletal and multi-organ staging, and assessment of response to therapy, with diagnostic performance superior to that of radiographs, BS, and CT.

The choice between WB-MRI and PET/CT should be guided not only by their respective diagnostic performance, the availability of specific PET tracers adapted to the cancer, the cellular or molecular characteristics of the cancer, but also by radiation safety concerns and local availability.

## Abbreviations

ADC: Average diffusion coefficient
BS: Bone scintigraphy
CT: Computed tomography
DL: Deep learning
DWI: Diffusion-weighted imaging
FDG: Fluorodeoxyglucose
FF: Fat fraction (fat fraction calculated from a Dixon sequence)
FSE: Fast spin echo
FO: Fat only (image of fat only from a Dixon sequence)
GE: Gradient echo
IP: In-phase
MRI: Magnetic resonance imaging
MGUS: Monoclonal gammopathy of undetermined significance
MM: Multiple myeloma
NF: Neurofibromatosis
OP: Out-of-phase
PSMA: Prostate-specific membrane antigen
PET/CT: Positron emission tomography with computed tomography
STIR: Short-time inversion recovery
WB-MRI: Whole-body MRI
WO: Water only (image of water only from a Dixon sequence)
